# Phagocytosis

**Published:** 1892-04-02

**Authors:** 


### The Hospital

An Institutional Journal of

Science, Medicine, Nursing, and Philanthropy.

*For Hospitals, Asylums, Infirmaries, Dispensaries, Medical Practitioners, Students, Nurses, and the Charitable Public.*

Vol. XII.—No. 288.] [April 2, 1892.

### PHAGOCYTOSIS.

It used to be said in politics that what was proposed in Manchester one day would be accepted throughout England the next. A similar state of things is coming about with regard to the relations between scientific medicine and the general public, so that we may almost now say that what engages the attention of the physiological laboratory this year will be read with interest by every newspaper subscriber in the world during the next. It is hardly necessary to explain, even to non-professional readers, what "phagocytosis" means. But as we look on with the eyes of a steady imagination at the wonders which Continental and native physiologists have laid open to our view, we become gradually conscious of a world of fact and of law which is destined to profoundly modify every common intellectual conception of the present and future generations.

Observant persons have long known that we live in a world of astonishing vitality. Living forms, of almost every conceivable variety, surround us everywhere. They float in the air; they have their habitat on the solid earth; they crowd the sea. But the world at large has not hitherto been in the habit of considering every individual man as a kind of small universe in himself. Yet such is the fact. Within us, being born, living, performing their almost conscious life-work, dying and being reduced once more to their elements, are thousands, millions of small organisms, so small that our unassisted eyes cannot even discern their presence, and yet so truly cosmical that even the fully-developed, intellectual man is not more the subject of law, fixed order, and eternal necessity than they are.

How does all this concern the practical man? Let us ask another question: How do trifles like pennies concern the accountant of the millionaire? Sound finance on a large scale is not possible without the daily correct management of infinitesimal details. In the same sense, it is indispensable that the practical man of physiology and of physic shall know the internal and minute history of every day's life and activity in every part of the human organism. At the present moment the facts of "phagocytosis" are to many of us merely curious, and to some even oppressive as well. What are we going to do with all this strange new knowledge? We are told, on what seems indisputable authority, that when a fluid containing bacteria, such, for example as vaccine lymph or any other virus, is injected into the animal body at a given point, there is an immediate mobilisation of the white cells of the blood at that spot, and an effort on the part of these cells to seize every bacterium thus introduced, and to kill and devour it. Supposing that this statement of the case is actually true and correct, and it seems to us to be so, Dr. Klein and Professor Burdon Sanderson notwithstanding, how does it help us in actual every day medicine? So far as one can see the most practical thing to be done, on the discovery of such facts, would be to vastly increase the standing armies of Phagocytes which the human body contains, in numbers and efficiency. But can this be done? Have we any drugs, or any foods, or any kinds of physical discipline which will increase the phagocytes so much in numbers and efficiency that they shall be able to cope at once and successfully with every kind of bacteria that are injurious to our well being? If any means and methods were known which would justify us in answering this question in the affirmative, then typhoid fever, diphtheria, scarlet fever, small-pox, hydrophobia, consumption, and in short every form of disease of bacterial origin might be vanquished forthwith.

But so far as we know at present there are no known means of very greatly increasing the number and efficiency of the phagocytes of the human body. What is more, a too great preponderance of leucocytes over the red cells in the blood always appears to be a cause, or at least an evidence, of disease. There is here a strict parallelism with a too large and too costly standing army in any given State. When a disproportionate number of men are drawn from peaceful pursuits to be converted into soldiers, there are at least two evils which invariably result; agriculture and other necessary industries are injuriously depleted, and the interests which are thus rendered incapable of high productiveness are still further weakened and injured by being excessively taxed for the support of the unduly large standing armies. So far as we know at present the internal politics of the human body are somewhat analogous to this condition of things. The leucocytes, which play the part of defenders against bacterial invasions, cannot be increased beyond certain more or less definable limits; and if, which sometimes happens as the result of abnormal processes, they are unduly increased, the increase itself, or its associated condition, is not advantageous but mischievous.

Where are we, then, in our physiological explorations? Are we advancing on lines that will bring us manifestly beneficial results, or is the only fruit of our knowledge to be a keener vision for the dangers that surround us, with no increase of power to avert them? Whatever other practical result may spring from physiological exploration, it is certain that men are becoming far more conscious of the dangers that lie in wait for them. Moreover, this consciousness of danger will become increasingly intensified, because the physiologists constitute one of the most determined and intrepid armies the world has ever seen. They must, and will, have knowledge. The inward hunger to know is a passion, an enthusiasm, even a religion.

All the world demands "cures": it will not give either money or admiration for anything but cures. Now, the physician of sound intelligence, who is not a mere trader, regards prevention as of a thousand times more real value than cure. This judgment is commonly expressed by the formula, "An ounce of prevention is worth a pound of cure." It would be much nearer the "true truth" of things to say that a single grain of prevention is worth a ship-load of cure. And yet the preventionist is considered of less moment than the inventor of a "cure-all" pill. It cannot surely be such a very difficult thing to see that prevention is gold, whilst cure is but bronze. Whether is better—a hooped tree, whose trunk has been split by lightning, or a grand forest giant which the lightning has never touched? The cleverest plumber who puts on a hoop cannot compare with the man of genius who turns the lightning aside so that it never strikes the tree at all. What we are to look for in the physiological laboratory is not this or that method of cure only, but all kinds of knowledge which will enable us to prevent disease. This idea of prevention is far more modern, and excites far more enthusiasm in the truly scientific mind than the cutting and carving of the skilful surgeon, or the pilling and powdering of the prescribing physician. Cutting surgeons we must have, and prescribing physicians too. But the medical millennium which every scientific practitioner looks forward to is the golden age when all preventable diseases shall be extirpated root and branch, and when natural failures and decays shall be reduced to such easy gradations that the approach of death shall hardly be so much as perceived.

Phagocytosis, from a disease-prevention point of view, is a gain of knowledge of a fundamentally important kind. It simplifies some medical difficulties by showing us the strength and weakness of opposing forces. We see now that it is impossible for phagocytal armies always to vanquish the various bacterial hosts that may invade the human body. This one fact is made very clear to us, that we must not look to our defences alone if we hope to win successfully through a long life struggle. The defences are good so far as they go, and they can be strengthened somewhat by wholesome living, but they are not of themselves sufficient. The directing brain of man must look elsewhere than to his phagocytes for the means of victory over physiological enemies. He must attack those enemies, not when they are at his gates only, but in their own strongholds, by armies other than phagocytes. In a word, isolation hospitals, perfect drainage, the rational disposal of sewage, the purification of drinking waters and of all waters, and the equally important purification of the atmosphere in which we live, with other methods of a real sanitation, and possibly of inoculation, are the lessons which a knowledge of phagocytosis impresses upon us. These may seem to the general reader to be but counsels of perfection. But it is with physiology, as with other kinds of knowledge—the abstruse difficulties of to-day will be the scientific commonplaces of to-morrow.

